# Agressiveness in a patient diagnosed with Huntington’s Chorea hospitalized in a psychiatry Unit

**DOI:** 10.1192/j.eurpsy.2026.11614

**Published:** 2026-07-31

**Authors:** F. Pascual Pinazo, P. Nava García, M. E. Arevalo Gil, M. Sobredo Vega, S. P. Orrego Molina, E. Luján Pérez, A. Ibañez Ballesteros

**Affiliations:** 1 Psiquiatría, Hospital Infanta Leonor, Madrid; 2 Psiquiatría, Hospital del Sureste, Arganda del Rey, Madrid, Spain

## Abstract

**Introduction:**

Huntington´s chorea is is a fatal neurodegenerative disease, mostly inherited, that presents psychiatric, cognitive, and motor symptoms, commonly become noticeable between the ages of 30 and 50 years. No cure for Huntington´s desease is known, and full-time care is required in the later stages.

**Objectives:**

To describe a clinical case of a woman with no personal psychiatric history that presents agressiveness of unknown origin

**Methods:**

Case report and brief literature review of behavioral syptoms in Huntington´s Chorea

**Results:**

A 40-years-old woman is hospitalized in the psychiatry unit transferred from the police station. With an amazing lack of emotion, she tells that her seven-year-old son was crying because he didn’t like the food, and then she threatened him with a knife and stabbed it in his leg. Fortunately, no severe damages were done to the child. During her hospitalization, she always says “It wasn’t bad that”, and she doesn’t ask for forgiveness.

Her familiy name is very strange and infrequent; and we remember that another woman with the same family name was diagnosed with Huntington’s Disease three years ago. When we find out they are sisters, we make the genetic tests that confim the Huntington´s Chorea.

**Conclusions:**

In this case, it was easy to think in Huntington’s Chorea as the cause of agressiveness; but it is very important to make a complete clinical history, including the familiy members, in every patient, specially in those with no accurate diagnosis.

In adition, genetic counseling is highly recomended to prevent the disease in offspring.

**Disclosure of Interest:**

None Declared

